# Tropomyosin in mugwort cross-reacts to house dust mite, eliciting non-Th2 response in allergic rhinitis patients sensitized to house dust mite

**DOI:** 10.1186/s12948-021-00142-z

**Published:** 2021-04-02

**Authors:** Su Duan, Limin Zhao, Yuling Zhang, Nan Zhang, Ming Zheng, Qiqi Wang, Xu Zhang, Xiangdong Wang, Sun Ying, Claus Bachert, Luo Zhang, Feng Lan

**Affiliations:** 1 Beijing Key Laboratory of Nasal Diseases, Beijing Institute of Otolaryngology, Beijing, China; 2 Department of Otolaryngology-Head and Neck Surgery and Department of Allergy, Beijing Tongren Hospital, Capital Medical University, Beijing, China; 3 Upper Airways Research Laboratory, ENT Department, Ghent University, Ghent, Belgium; 4 Department of Immunology, School of Basic Medical Science, Capital medical University, Beijing, China

**Keywords:** Cross-reactivity, House dust mite, Mugwort

## Abstract

**Background:**

Mugwort and house dust mite (HDM) are two of the most common inhalant allergens in Asia, however, whether mugwort affects polysensitized HDM^+^ allergic rhinitis (AR) patients has not been elucidated.

**Methods:**

Overall, 15,884 AR outpatients were assessed for clinical status. Amino acid sequences of mugwort were determined by mass spectrometry. Afterward, cross-reactivity between mugwort tropomyosin and Dermatophagoides pteronyssinus 10 (Der p10) was analysed by ELISA inhibition and basophil activation experiments. To compare immunologic responses eliciting by two different tropomyosins, peripheral blood mononuclear cells (PBMCs) of HDM-monosensitized patients were stimulated by mugwort, HDM, Der p10 and synthetic peptides representing mugwort tropomyosin respectively.

**Results:**

Polysensitized HDM^+^AR patients were mainly sensitized to cat and mugwort, and the positive rate of monosensitized HDM^+^AR out-clinic patients was increased during the mugwort pollen season. Tropomyosin protein was able to find in mugwort. Synthetic tropomyosin peptide of mugwort activated basophils which were primed by HDM-specific IgE; ELISA inhibition experiment showed synthetic tropomyosin peptide of mugwort inhibited IgE binding to HDM tropomyosin, Der p10. Unlike HDM and Derp 10, mugwort and mugwort tropomyosin mainly induced IFN-γ and IL-17 release in PBMCs of monosensitized HDM^+^AR patients, but not IL-5.

**Conclusions:**

Pan-allergen tropomyosin accounts for the cross-reactivity between mugwort and HDM, which reminds HDM^+^ patients to reduce mugwort exposure in mugwort pollen season in virtue of the tropomyosin induced mild inflammation.

**Supplementary information:**

The online version contains supplementary material available at 10.1186/s12948-021-00142-z.

## Introduction

Allergic rhinitis (AR), an upper airway allergic inflammatory disease, causes symptoms of sneeze, runny nose, nasal obstruction and itchy nose, which is predominantly mediated by type 2 helper (Th2) cells and immunoglobulin E (IgE) [1, 2]. Among the common triggering allergens, house dust mites (HDM), mould spores and animal dander mainly cause symptoms of perennial AR, whereas, a large variety of pollens from different geographical regions contributes to symptoms of seasonal AR [3]. Some AR patients are found to be polysensitized to more than one allergen [4], and an increasing number of sensitizations strongly predisposes AR patients to allergic asthma [5, 6]. Thus, the treatments for polysensitized AR patients closely associate with asthma management [7].

Allergen specific immunotherapy (AIT) is an effective therapeutic method for monosensitized AR patients [7]. However, management approaches to polysensitized AR patients by AIT are not standardized yet. There are intercontinental differences in allergen products available for AIT in polysensitized patients [8]. Desensitization to the most clinically relevant allergen is often used to treat polysensitized patients in Europe and in China, while mixtures of extracts are recommended in the United States [9, 10]. Differences in therapeutic effects of single AIT have been shown, in which more effective in reducing the symptoms are observed in those of monosensitized patients than that of polysensitized patients treated with the same dose [11, 12]. However, there are no obvious change in HDM-specific IgE production and a lower concentration of HDM-specific IgG4 in polysensitized patients compared with those of monosensitized patients after AIT [12, 13]. Polysensitization is mainly caused by cross-reactivity among closely related allergens, or allergens from other sources. Thus, the identification of primary causal allergen(s) and sensitization to cross-reacting allergens could help us to find efficient ways for treating polysensitized AR patients in the near future.

HDM and mugwort have been regarded as the two most common and clinically relevant sensitizing allergens in AR patients in Asia [14]. HDM cross-reacts with allergens from other invertebrates, including other species of mites, insects, mollusks, and crustaceans [15]. It is not clear whether or not there is cross-reactivity between HDM and mugwort; consequently, whether or not mugwort affects polysensitized HDM^+^AR patients. In view of this, the present study has specifically investigated cross-reactivity between HDM and mugwort in HDM^+^AR patients.

## Methods

### Study design and subjects

Subjects with AR based on criteria of the AR and its Impact on Asthma (ARIA) consensus statement [16] were recruited consecutively from the allergy-rhinology outpatient clinic of Beijing Tongren Hospital. On recruitment, each subject completed a questionnaire to record demographic data, nasal symptom severity, and history of asthma; and blood samples were collected from each subject for analysis of serum specific IgE antibodies. Peripheral mononuclear cells (PBMCs) were also prepared from blood samples of some healthy controls and HDM monosensitized AR patients. Because there was lack of a reliable validated assay, whether HDM monosensitized AR patients had IgE-reactivity to Der p10 were unknown. None of the subjects had received any allergen-specific immunotherapy or monoclonal antibody treatment. The study was approved by the Medical Ethics Committee of Beijing Tongren Hospital, and all patients provided written informed consent before entry into the study and collection of any samples.

### Serum antigen-specific IgE measurements

The presence of IgE antibodies in blood was determined using a EUROLINE Atopy Screen (DP 3713 E; Lubeck Germany), which comprised two sets of allergens; one with a mix of aeroallergens [including tree mix (willow, poplar, elm), common ragweed, mugwort, house dust mite mix (Dermatophagoides pteronyssinus (Der p), Dermatophagoides farinae (Der f)), house dust, cat, dog, cockroach German, mould mix (*Penicillium notatum*, *Cladosporium herbarum*, *Aspergillus fumigatus*, Alternaria alternata) and hops], and one with a mix of food allergens [including egg white, cow’s milk, peanut, soybean, beef, mutton, sea fish mix (codfish, lobster, scallop), shrimp, and crab]. Furthermore, concentrations of Der f2 specific IgE, Der p1 specific IgE, and total IgE were also measured using the ImmunoCAP system (Immunodiagnostics; Thermo Fisher Scientific, Uppsala, Sweden). Allergen-specific IgE > 0.35 kU/L was considered as positive.

### Mugwort protein analysis by mass spectrometry

Prior to analysis, 100 mg samples of mugwort (*Artemisia sieversian* (*A. sieversian*)) were separately prepared as peptide solutions by denaturing and treatment with protease trypsin according to the method described by León and colleagues [17]; and then analysed in a Triple-TOF 6600 mass spectrometer (Sciex, United States) fitted with a Nanospray III source (Sciex). The ion spray voltage was 2300 V, declustering potential 80 V, curtain gas 35 psi, nebulizer gas 5psi, and interface heater temperature at 150 °C. The peptides were introduced into the mass spectrometer via Nona 415 liquid chromatography column (Sciex) eluted with water/acetonitrile/formic acid (buffer B: 2/98/0.1%). In this regard, samples (4 μL) were injected onto a C18 desalted column (3 μm, 120 Å, 350 µm × 0.5 mm), and separated onto a C18 analysis column (3 μm, 120 Å, 75 µm × 150 mm) with gradients ranging from 5 to 16% buffer B in the first 25 min, from 16 to 26% buffer B in the next 20 min, from 26 to 40% buffer B in the following 3 min, from 40 to 80% buffer B in the next 5 min, and finally from 80 to 5% buffer B in the final 7 min; at a flow rate of 0.6 μL/min. Since the genome sequence annotation database of *Artemisia sieversian* are unavailable, the peptides presented in the samples were matched to the UniProt *Artemisia* carvifolia databases. All identified corresponding proteins in *A. sieversian* were separately listed in Additional file 1: Table S1.

### Synthesizing tropomyosin peptide of mugwort

According to the result of mass spectrometry, two common repeat peptide sequences of tropomyosin protein from *A. sieversian* were synthesized from SynPeptide company (Shanghai, China) as follows: VGSPDESYEDFTNSLPSNECR; IEEQQVIVEK. Giving the preliminary data of ELISA inhibition experiment, basophil and PBMC activation experiments, synthetic peptide with sequence of VGSPDESYEDFTNSLPSNECR was chosen as the representative sequence for tropomyosin protein from mugwort.

### HDM-specific IgE blockage by synthetic peptide of mugwort tropomyosin

Serum samples of 15 HDM^+^AR patients with a high or low level of HDM-specific IgE were used to assess whether the IgE can be pre-blocked by synthetic peptides of mugwort tropomyosin. Briefly, 200 μL of serum from HDM^+^AR patients were incubated with or without synthetic mugwort tropomyosin peptides (1000 ng/mL for each) for 1 h at room temperature, and at the end of incubation the serum samples were analysed for the concentrations of HDM-specific IgE using the ImmunoCAP system.

### Basophil activation test

PBMCs isolated from non-allergic donors (5 $\times$ 10^5 cells) were stripped in 2 mL ice cold lactic acid buffer (0.13 M KCl, 0.05 M NaCl, 0.01 M lactic acid, pH = 3.9) for 30 s as described before [18]. After washing 3 times by PBS, cells were pre-incubated with sera from HDM-allergic individuals for 1 h at 37 °C. And then, cells were stimulated by different concentrations of synthetic mugwort tropomyosin or Derp 10 (50, 500 ng/mL) in hepes buffer containing IL-3 (R&D, Minneapolis, Minnesota, USA). In the meanwhile, cells exposed to FLMP (Sigma, St. Louis, USA)) were taken as a positive control. The reaction was stopped by EDTA buffer (20 mM). In the end, PBMCs were stained with basophil surface markers: CD123BV650, CCR3-APC-fire750 and CD63-PE (BioLegend, San Diego, CA, USA), and the percentages of CD63^+^CD123^+^CCR3^+^ cells were analysed by Flowjo software.

### ELISA inhibition experiment

Plates were pre-coated with Der p10 obtained from CUSABIO (Wuhan, China) overnight at 4 °C, then incubated with PBS supplemented with 1% BSA and 0.05% tween 20 for 6 h at room temperature to reduce non-specific binding. Inhibition was performed by adding sera from HDM monosensitzed patients with synthetic mugwort tropomyosin peptides (50, 500 ng/mL), and sera without peptides were taken as non-inhibition conditions. Anti-human IgE (2 μg/mL, NOVUS, USA) were added, followed by streptavidin-HRP conjugated secondary antibodies (diluted 1:2000; EasyBio, Beijing, China). Absorbance was determined using an ELISA reader (BioTek, Vermont, USA) at 405 nm. All experiments were performed in duplicate. Percent inhibition was calculated using the following equation: percent inhibition = 100 − [(OD of serum with tropomyosin peptide/OD of serum without peptide) × 100].

### Stimulation of PBMCs ex vivo

PBMCs were isolated from the blood of 6 healthy donors, 16 HDM^+^AR patients and 1 mugwort^+^ AR patient using Ficoll-Hypaque density gradient centrifugation according to the standard protocol (Lymphoprep™, Nycomed Pharma, Oslo, Norway). Cells were plated at a density of 1 × 10^6^ cells/well in a 24-well plate in 0.5 mL RPMI 1640 (Gibco, USA) culture medium containing either HDM (Der p1 extract; 0.2, 1, 5 μg/mL; GREER Laboratories, Lenoir, NC, USA), mugwort (1, 10, 100, 1000 ng/mL; *A. sieversian* locally prepared in Beijing Tongren Hospital), or synthetic peptides of mugwort tropomyosin, and then incubated at 37 °C in 5% CO_2_ for 48 h. Cells incubated with RPMI 1640 medium alone were used as controls. After incubation, the cell suspensions were collected and the supernatants were assessed for IL-5, IL-17, and IFN-γ using Luminex xMAP suspension array technology in a Bio-Plex 200 system (Bio-Rad, MI). All cytokine kits were purchased from R&D Company and the results were expressed as pg/mL.

### Statistical analysis

Statistical analysis was performed using the SPSS version 22.0 software package (IBMCorp, Armonk, NY, USA). Categorical variables were described using frequencies and/or percentages and continuous variables were presented as mean ± standard deviation (SD). Multiple logistic regression was used to analyse the possible risk factors for polysensitized HDM^+^AR patients. The influence of polysensitization on asthma development was assessed by the Chi squared test. The prevalence of different allergens in HDM^+^AR patients was estimated using Fisher’s exact test and logistic regression. The Wilcoxon test was used for paired comparisons of the effect of specific antigen stimulation on the release of cytokines from PBMCs, and the effect of synthetic mugwort peptides on blocking HDM specific IgEs between groups. *P* values of less than 0.05 were regarded as statistically significant.

## Results

### Mugwort affected the prevalence of HDM^+^AR patients

**Fig. 1:**
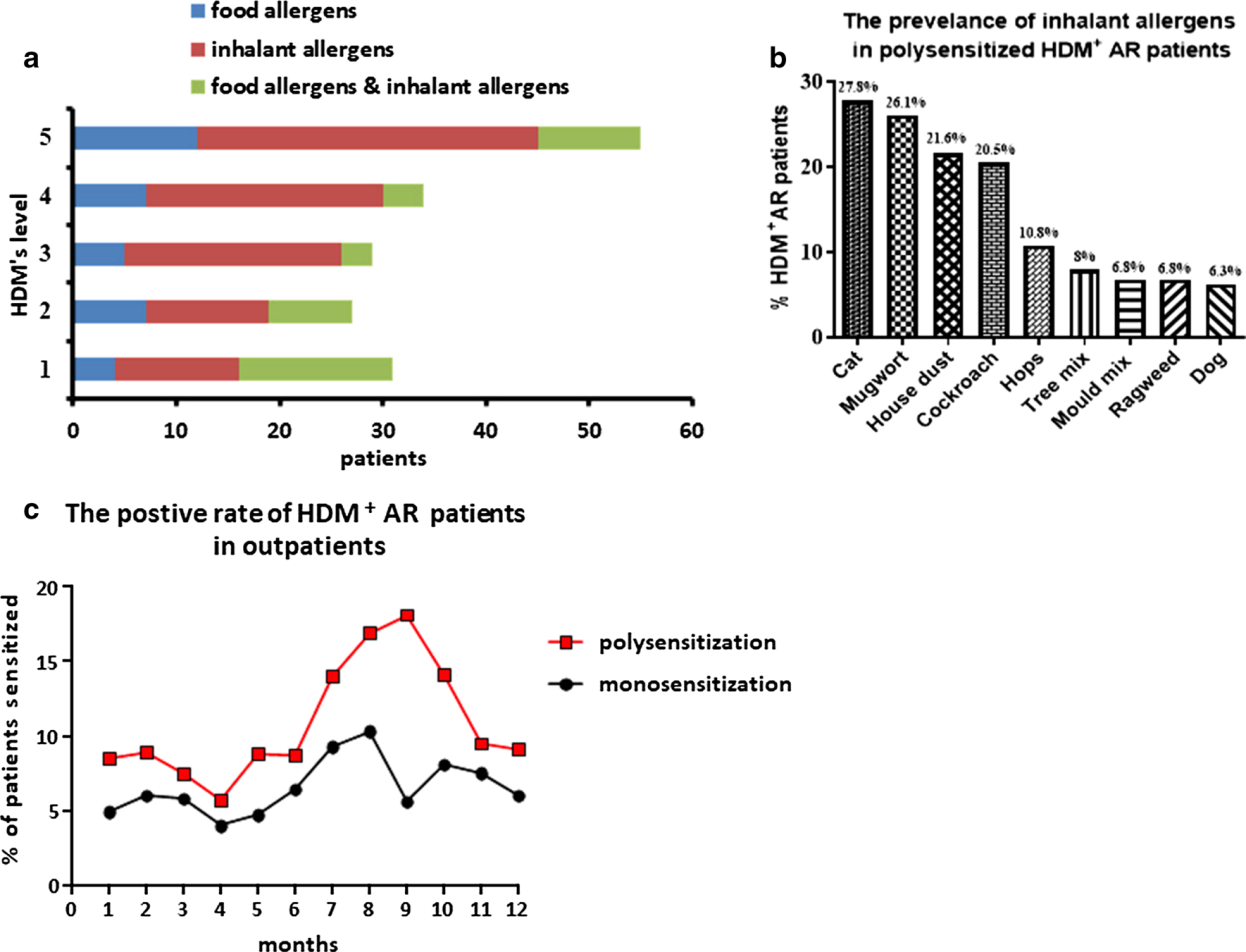
**a** The distribution of allergen types, according to the concentrations of house dust mite (HDM) specific IgE in polysensitized HDM^+^ allergic rhinitis (AR) patients. Food allergens included crab, shrimp, soybean, sea fish mix 1, egg white, beef, cow’s milk, peanut and mutton; while inhalant allergens included cat, mould mix 1, mugwort, hops, common ragweed, dog, cockroach, German, tree mix 2 and house dust. **b** The prevalence of inhalant allergens in polysensitized HDM^+^AR patients (n = 176). **c** The positive rate of monosensitized and polysensitized HDM^+^AR patients in recruited outpatients from January 2018 to December 2018 (n = 15354)

A total of 497 HDM^+^AR patients were recruited into the study. Overall, 64.6% of the HDM^+^AR patients were monosensitized, and 35.4% polysensitized (Table 1). Comorbid asthma was more prevalent in 19.32% of all polysensitized AR patients compared to in 9.03% of those monosensitized AR patients (*p *= 0.001). Type of sensitizing allergens was further analysed in polysensitized HDM^+^AR patients in parallel with the HDM-specific IgE level. Regardless of the level of HDM-specific IgE detected, sensitization was greatest to inhalant allergens in the polysensitized HDM^+^AR patients (Fig. 1a). The five most prevalent inhalant allergens in the polysensitized HDM^+^AR patients were cat (27.8%, 95% CI 21.2–34.5%), mugwort (26.1%, 95% CI 19.6–32.7%), house dust (21.6%, 95% CI 15.5–27.7%), cockroach (20.5%, 95% CI: 14.4–26.5%) and hops (10.8%, 95% CI 6.2–15.4%) (Fig. 1b). The number of monosensitized HDM^+^AR patients was increased from July to August, while the increased number of polysensitized HDM^+^AR patients was from July to September (Fig. 1c), which appeared to follow the trend of the mugwort pollen season seen from July to early September in 2018 [19].

**Table 1:** Demographic and clinical characteristics of house dust mite-positive allergic rhinitis patients investigated

	Monosensitization (n = 321) (64.4%)	Polysensitization (n = 176) (35.4%)	*P* value	Odds ratio	95% CI
Gender			0.058	0.690	0.470–1.012
Male	158 (49.22)	101 (57.39)			
Female	163 (50.78)	75 (42.61)			
Age	29.60 ± 11.55	28.77 ± 12.09	0.449		
Family history of AR	69 (21.50)	54 (30.68)	0.056	1.522	0.989–2.342
Smoking and drink	222 (69.16)	99 (56.25)	0.003*	0.560	0.381–0.823
Co-morbid Allergic status					
Asthma	29 (9.03)	34 (19.32)	0.001*	2.486	1.448–4.267
Atopic dermatitis	28 (8.72)	24 (13.64)	0.118	1.506	0.818–2.772
Allergic conjunctivitis	34 (10.59)	17 (9.66)	0.206	0.648	0.331–1.269
HDM specific IgE (kU/L)	25.44 ± 26.66	29.89 ± 29.92	0.112	1.005	0.999–1.012

HDM: house dust mite

*P < 0.05

### Tropomyosin was involved in mugwort-HDM cross-reactivity

The genome sequence annotation database of mugwort is still unavailable, thus peptide amino acid sequences of *A. sieversian* were matched to the UniProt *Artemisia* carvifolia databases, which indicated the presence of cross-reactivity protein tropomyosin (Table 2). Similarly, the presence of cross-reactivity proteins profilin and lipid transfer protein were also found in *A. sieversian* (Additional file 1). Due to lack of a whole protein amino acid sequence of tropomyosin in mugwort, we used synthetic tropomyosin peptide of mugwort instead.

**Table 2:** Amino acid sequences of *Artemisia sieversian* tropomyosin fragments detected by mass spectrometry

Mugwort species	Names	Conf. %	Sequence
*Artemisia sieversian*	Actin-binding, cofilin/tropomyosin type	99	IEEQQVIVEK
	Actin-binding, cofilin/tropomyosin type	99	VGSPDESYEDFTNSLPSNECR

**Fig. 2:**
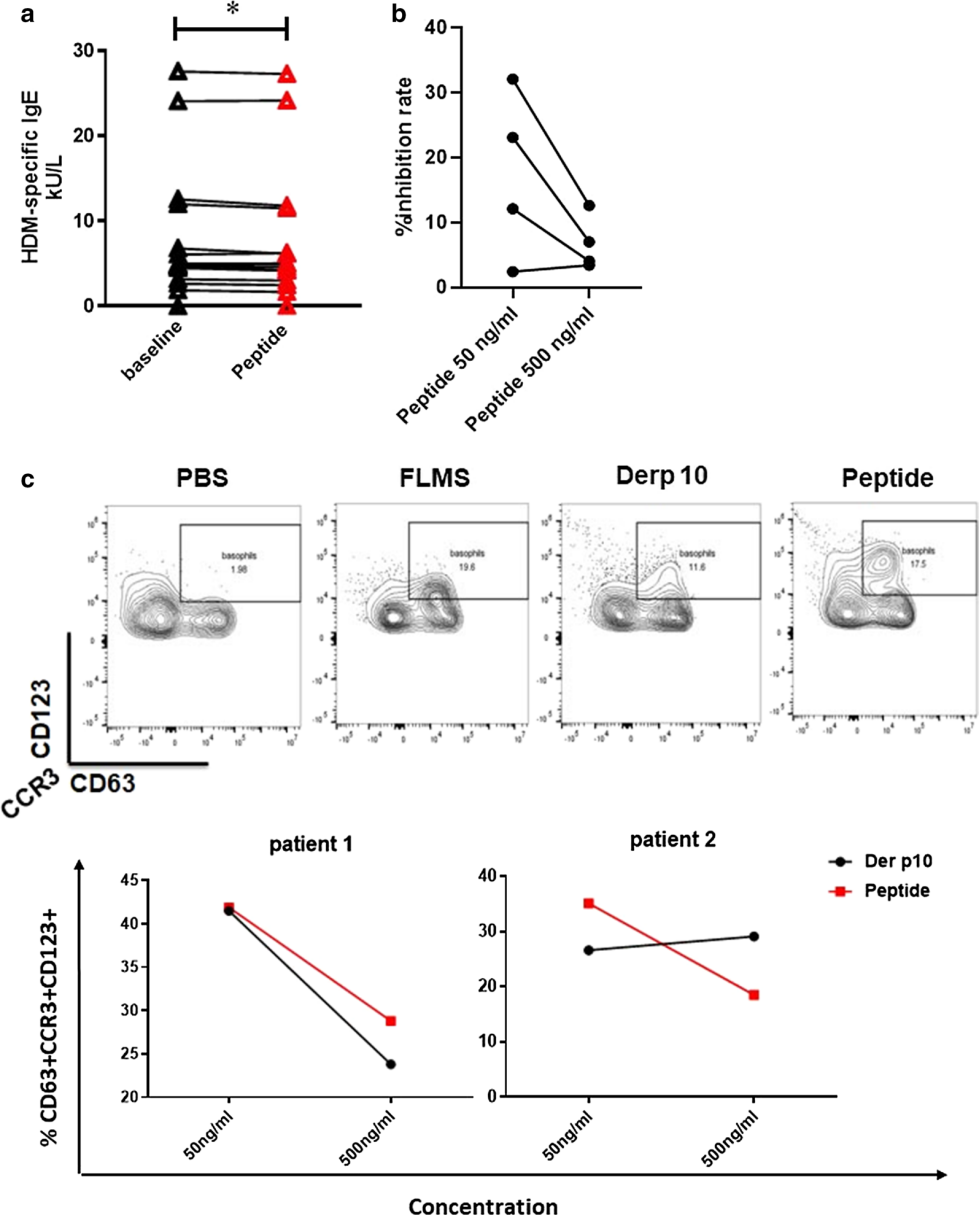
**a** Concentrations of house dust mite (HDM)-specific IgE in the serum of HDM^+^ allergic rhinitis (AR) patients (n = 15) incubated in the absence or presence of synthesized tropomyosin peptides of mugwort; **b** Synthetic tropomyosin peptide of *Artemisia sieversian* (*A. sieversian*,) inhibits IgE-binding to Dermatophagoides pteronyssinus 10 (Der p10) measuring by ELISA. Tropomyosin of mugwort exhibits inhibitory effect in 4 out of 8 patients at the concentration of 50 ng/mL. **c** The expression of basophils (CCR3^+^CD123^+^CD63^+^) with stimulations of Der p10 and synthetic tropomyosin peptide of mugwort. Totally, HDM specific IgE pre-incubated basophils from 2 out of 8 patients were activated by tropomyosin of mugwort

Pre-incubation serum samples of monosensitized HDM^+^AR patients with synthetic tropomyosin peptide of mugwort significantly decreased the concentrations of HDM specific-sIgE in the serum (Fig. 2a). Furthermore, tropomyosin peptide of mugwort (50 ng/mL) inhibited IgE binding to Der p10 ranging from 2.4% to 32.1% (Fig. 2b). As shown in Fig. 2c, in the presence of Der p10 and mugwort tropomyosin peptide, the activation of basophils pre-sensitized by HDM-specific IgE occurred in 2 out of 8 non-allergic patients.

### Mugwort and synthetic tropomyosin peptide of mugwort induced non-Th2 response in PBMCs of monosensitized HDM^+^AR patients

**Fig. 3:**
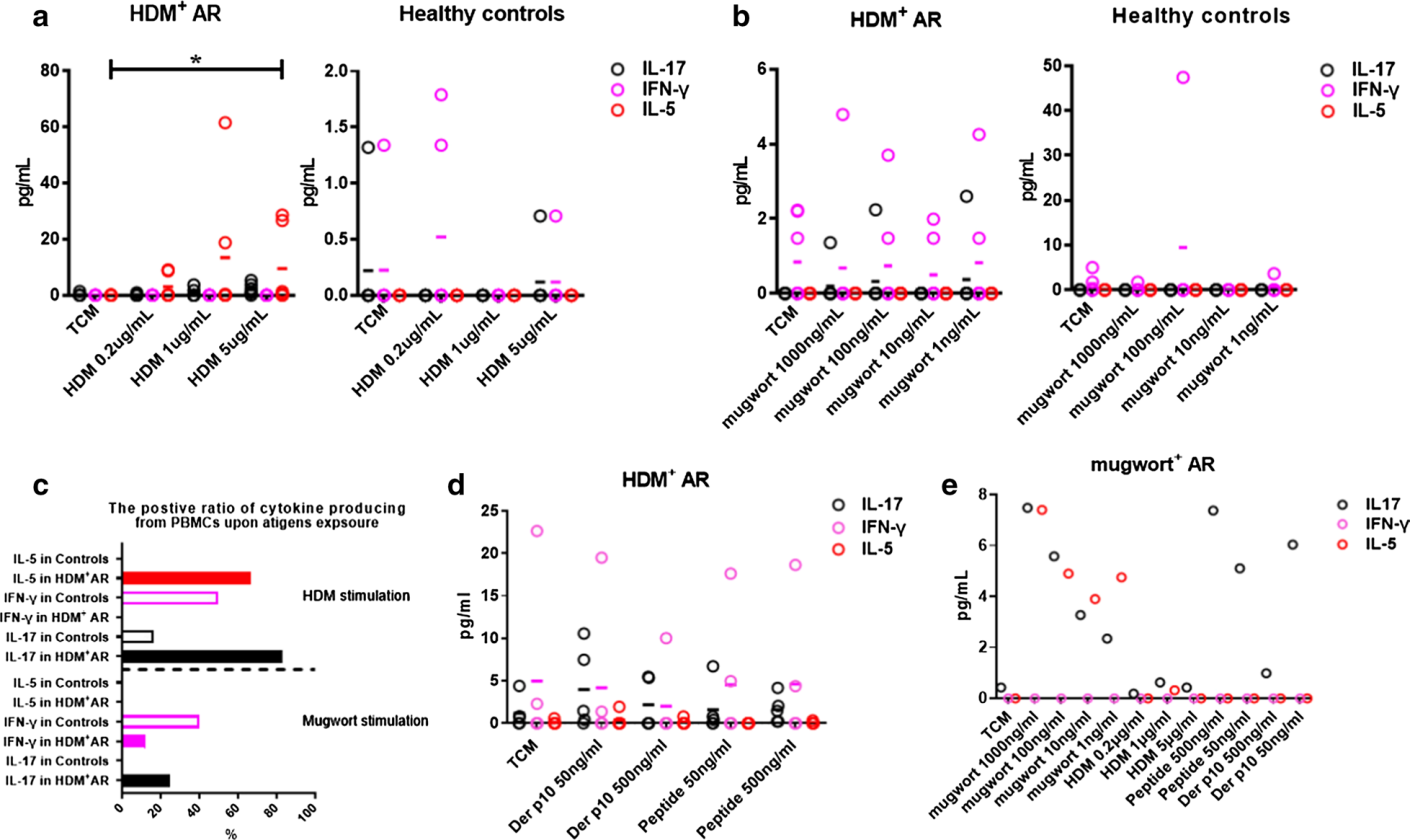
Effect of specific antigen on release of IL-17, IL-5 and IFN-γ in peripheral blood mononuclear cells (PBMCs) of HDM^+^ allergic rhinitis (AR) patients or healthy controls. **a** Stimulation of PBMCs of monosensitized HDM^+^AR patients and control subject with house dust mite (HDM) (n = 6); **b** Stimulation of PBMC of monosensitized HDM^+^AR patients (n = 8) and control subjects (n = 5) with mugwort; **c** The positive ratio of cytokine produced by PBMCs of monosensitized HDM^+^AR or controls upon HDM and mugwort stimulation. **d** Concentrations of IL-17, IL-5 and IFN-γ released in PBMCs from HDM^+^AR patients stimulated with synthesized tropomyosin peptides of mugwort and Der p10 (n = 4). **e** Concentration of IL-17, IL-5 and IFN-γ released in PBMCs of mugwort^+^AR patients with mugwort, synthetic tropomyosin peptide of mugwort, house dust mite (HDM), tropomyosin of HDM Der p10 stimulations (n = 1)

Compared to medium controls, HDM stimulated PBMCs isolated from monosensitized HDM^+^AR patients (n = 6) to produce high levels of IL-5 (0.59–61.56 pg/mL) and IL-17 (0.91–5.27 pg/mL), but not IFN-γ (Fig. 3a). In contrast, HDM induced PBMCs isolated from healthy controls (n = 6) to release IL-17 (0.71–1.32 pg/mL) and IFN-γ (0.71–1.79 pg/mL), but not IL-5 (Fig. 3a). Interestingly, mugwort stimulated PBMCs from monosensitized HDM^+^AR patients (n = 8) to produce IL-17 (1.36–2.24 pg/mL) and IFN-γ (3.7–4.79 pg/mL), but not IL-5 (Fig. 3b); while such stimulation only induced the production of IFN-γ (1.79-47.45 pg/mL), but not IL-5 or IL-17 by PBMC of healthy controls (n = 5) (Fig. 3b). Generally, the frequency of mugwort-induced release of IL-17 and IFN-γ was 25% and 12.5%, respectively, from PBMCs of monosensitized HDM^+^AR patients; and the frequency of HDM-induced release of IL-5 and IL-17 was 66.7% and 83.3%, respectively, from PBMCs of monosensitized HDM^+^AR patients (Fig. 3c). This suggest that HDM is more effective than mugwort in inducing inflammation in HDM^+^AR patients.

To confirm that tropomyosin may be responsible for mugwort extract-induced non-Th2 response in monosensitized HDM^+^AR patients, PBMCs from those patients with monosensitized HDM^+^AR patients were incubated with tropomyosin peptides of mugwort and Der p10. In fact, mugwort tropomyosin peptide induced the synthesis and release of IFN-γ, with about 20% frequency and 60% of subjects released IL-17, whereas the frequency of IL-5 and IL-17 induced by Der p10 was 20% and 60% respectively (Fig. 3d). Like mugwort tropomyosin, Der p10 mainly induced the release of IL-17 in PBMCs of mugwort^+^ AR patients (n = 1) (Fig. 3e).

## Discussion

We demonstrated for the first time that cross-reactive protein tropomyosin in mugwort and HDM is responsible for the cross-reactivity between HDM and mugwort. However, unlike HDM and Derp 10, mugwort and synthetic tropomyosin peptide induced Th1 and Th17 in PBMCs of HDM monosensitized AR patients, but not Th2 response.

Tropomyosin, a pan-allergen, belongs to a family of phylogenetically conserved proteins with multiple isoforms present in muscle and non-muscle cells of vertebrates and invertebrates [20]. It has been known that tropomyosin from HDM and cockroaches share high sequence homology with that of shellfish, which unsurprisingly results in cross-reactivity among HDM, cockroach and food allergens [21–23]. Mugwort is the most important outdoor seasonal allergen in Asia [4, 24]. Our data have shown that there is a large number of polysensitized HDM^+^AR patients who are sensitized to mugwort. Furthermore, the number of monosensitized HDM^+^AR patients is increased from July to August. Mite densities indeed vary with seasons and areas. Reportedly, three peaks for the domestic mites density in Beijing appear in September to October, January and May [25]. Thus, these suggest that the increased number of monosensitized HDM^+^AR patients in July and August might likely be affected by mugwort. Although amino acid sequences of mugwort (*A. sieversian*) tropomyosin proteins are different from that of HDM tropomyosin Der p10. Considering that the sequence of the same protein varies in different species and cross-reactivity is thought to occur when a protein of similar sequence, structure or family binds to T and B cell receptors [26]. Therefore, it is likely that tropomyosin might be involved in mugwort-HDM cross-reactivity due to tropomyosin in allergens.

The present study has indicated that stimulation of PBMCs of monosensitized HDM^+^AR patients with mugwort induced synthesis of IL-17 and IFN-γ, whereas stimulation with HDM induced synthesis of IL-5 and IL-17. Our group has previously demonstrated that single-nucleotide polymorphisms (SNPs) in IL-17A and IL-17F gene regions are potentially associated with the development of AR and comorbid asthma in Chinese subjects [27]. Similarly, a study in Caucasian subjects has also demonstrated that there is an association between serum IL-17 and the severity of clinical symptoms in AR patients [28]. As mentioned above, the role of Th17 in the pathogenesis of AR cannot be excluded. Thus, the induction of IL-17 by mugwort from PBMCs in monosensitized HDM^+^AR patients may be associated with clinical symptoms of patients. In this study, the finding for Der p10-induced synthesis of IL-5 in monosensitized HDM^+^AR patients was in accordance with the findings of stimulation by HDM. In the meanwhile, stimulation synthesized tropomyosin peptide of mugwort could induce IL-17 and IFN-γ by PBMCs of HDM^+^AR subjects, suggesting that tropomyosin is responsible for the cross-reactivity between HDM and mugwort, eliciting non-Th2 response.

In conclusion, we have for the first time demonstrated that mugwort tropomyosin cross-react to Der p10, and therefore might play a role in eliciting a non-Th2 response in polysensitized HDM^+^AR patients in comparison to HDM. Since mugwort stimulation may be related to clinical symptoms of HDM sensitized patients in autumn pollen season, avoiding to expose to mugwort should be recommended to HDM^+^AR patients who cross-react to mugwort. Furthermore, therapeutic agents for targeting non-Th2 response may potentially alleviate symptoms of patients. Whether HDM^+^AR patients are benefit for mugwort AIT due to the present of cross-reactive protein tropomyosin still needs to be further investigated.

## Supplementary information

**Additional file 1: Table S1.** Identified corresponding proteins in *Artemisia sieversian*.

## Data Availability

Not applicable

## Abbreviations

AIT: Allergen specific immunotherapy
AR: Allergic rhinitis
ARIA: Allergic Rhinitis and its Impact on Asthma
*A. sieversian*: *Artemisia sieversian*
Th2: Type 2 helper
Der f: Dermatophagoides farinae
Der p: Dermatophagoides pteronyssinus
HDM: House dust mites
IgE: Immunoglobulin E
PBMCs: Peripheral mononuclear cells
